# Trial-by-trial feedback fails to improve the consideration of acceleration in visual time-to-collision estimation

**DOI:** 10.1371/journal.pone.0288206

**Published:** 2023-08-02

**Authors:** Marlene Wessels, Heiko Hecht, Thirsa Huisman, Daniel Oberfeld

**Affiliations:** Johannes-Gutenberg-Universität Mainz, Mainz, Germany; University of Helsinki, FINLAND

## Abstract

When judging the time-to-collision (TTC) of visually presented accelerating vehicles, untrained observers do not adequately account for acceleration (second-order information). Instead, their estimations only rely on vehicle distance and velocity (first-order information). As a result, they systemically overestimate the TTC for accelerating objects, which represents a potential risk for pedestrians in traffic situations because it might trigger unsafe road-crossing behavior. Can training help reduce these estimation errors? In this study, we tested whether training with trial-by-trial feedback about the signed deviation of the estimated from the actual TTC can improve TTC estimation accuracy for accelerating vehicles. Using a prediction-motion paradigm, we measured the estimated TTCs of twenty participants for constant-velocity and accelerated vehicle approaches, from a pedestrian’s perspective in a VR traffic simulation. The experiment included three blocks, of which only the second block provided trial-by-trial feedback about the TTC estimation accuracy. Participants adjusted their estimations during and after the feedback, but they failed to differentiate between accelerated and constant-velocity approaches. Thus, the feedback did not help them account for acceleration. The results suggest that a safety training program based on trial-by-trial feedback is not a promising countermeasure against pedestrians’ erroneous TTC estimation for accelerating objects.

## 1. Introduction

Precise timing is essential for successful interactions with moving objects. In sports, the goal is usually to make contact with an object, e.g., an approaching ball, at the right time. In road traffic, road users likewise can judge approaching vehicles but with the goal of avoiding collisions. A pedestrian can safely cross a road on which a vehicle is approaching only if the time remaining until the arrival of the vehicle at her position (time-to-collision, TTC) is longer than the time the pedestrian needs to cross the road. Previous studies have repeatedly shown that humans tend to overestimate the TTC for approaching objects that accelerate positively (i.e., the speed increases during the approach)–predominantly when the object is only visually presented [1–8]. As TTC estimation is systematically related to pedestrians’ road-crossing behavior (gap acceptance) [9], and TTC overestimations for vehicles at higher constant speeds translate into riskier crossing decisions [10, 11], it is thus likely that pedestrians will estimate the TTC of an approaching vehicle that accelerates positively to be longer than it actually is, and may decide to cross the road before the vehicle, although the remaining time for a collision-free crossing is too short. To reduce the risk of accidents caused by situations in which the TTC is often overestimated, suitable countermeasures are desirable. We have previously shown that the sound of a conventional accelerating vehicle can help to remove this bias in visual TTC estimation for accelerated approaches [8]. However, this benefit of added vehicle sound was strongly reduced for electric compared to conventional vehicles [7]. To not forego the benefit of electric vehicles to reduce noise pollution but maintain pedestrian safety, observers should be put in a position to judge vehicle acceleration based on visual information. In the present study, we therefore investigated whether a training in which participants are asked to focus on visual cues for acceleration and receive trial-by-trial feedback about their visual TTC estimation accuracy could be such a potential countermeasure. To do so, we implemented a highly realistic virtual reality experiment, of which the ecological validity can be considered relatively high. However, the generalizability to real-world situations should still be evaluated, as it has been shown that road-user behavior may differ between laboratory and real traffic environments [12, 13].

The overestimation of TTC for positively accelerating vehicles typically follows a specific characteristic: it increases with the vehicle’s acceleration and with actual TTC, while it decreases with the vehicle velocity at the moment of estimation. This pattern is readily explained by the relationship between the first-order TTC (*TTC1*), defined by the first-order equation of motion, and the actual or second-order TTC (*TTC2*) for an accelerating vehicle,

$$TTC1\left(t\right)=\frac{D(t)}{v\left(t\right)}=TTC2\left(t\right)+\frac{a∙{\left(TTC2\left(t\right)\right)}^{2}\;}{2v\left(t\right)},$$ (1)

where *D*(*t*) represents the instantaneous vehicle distance, *a* the (constant) acceleration and *v*(*t*) the instantaneous velocity at the moment of estimation *t*, and *TTC2* is specified (for a > 0) as $TTC2\left(t\right)=\frac{-v\left(t\right)+\sqrt{{v(t)}^{2}+2∙a∙D(t)}}{a}$. This so-called first-order approximation [14] occurs when observers consider the object’s distance and velocity (first-order motion) but do not account for the object acceleration (second-order motion), although both need to be considered for an accurate TTC estimation for an accelerating object. Consequently, *TTC1* for an accelerating object equals the actual TTC of an object approaching at a constant velocity, given the same velocity and distance of both objects at the moment of estimation. The second term on the right-hand side of Eq 1 represents the systematic estimation error. For an approaching, positively accelerating object (*v*(*t*) ≥ 0, *a* > 0, *TTC2*(*t*) > 0), this error term is positive, and thus *TTC1* exceeds the actual TTC (*TTC2*). If, however, the object travels at a constant velocity, the error term is of course 0 and *TTC1* equals *TTC2*.

The non-consideration of acceleration during TTC estimation may partially depend on the rather poor sensitivity of our visual system to detect dynamic speed changes [15–22]. Nonetheless, informative feedback on the deviation of the estimated from the veridical TTC, i.e., knowledge of results (KR), will alert observers that they commit serious estimation errors. If they realize the nature of these errors, namely that overestimation of TTC occurs predominantly during accelerated approaches, the quantitative KR feedback could lead to a shift of their attention towards the second-order motion information, and thus to more accurate TTC estimation for accelerated vehicle approaches, provided they can detect the acceleration. In case the low sensitivity to visual second-order information prevents a direct attention shift to acceleration, quantitative KR feedback might enable observers to exploit information or prior knowledge that is correlated with acceleration and can be detected. Such an optimized estimation strategy might explain findings showing that observers can capitalize on the information in natural acceleration of gravity [23–26]. In other contexts, untrained observers were able to incorporate KR feedback, which improved their performance in perceptual learning tasks [27–30]. The study that may come closest to answering the question for objects moving on surfaces of different friction, is an experiment conducted by Brenner et al. [31], who presented accelerating objects for < 1000 ms (see also [32]). Their participants were unable to benefit from the acceleration information predicted by the context when receiving trial-by-trial feedback. However, the short viewing times may not have been sufficient to notice and/or process the acceleration information.

We therefore chose a longer viewing time (2500 ms) that should clearly provide sufficient time to detect acceleration, if possible at all. We presented participants with constant-velocity and accelerated vehicle approaches at different TTCs. We first measured their estimated TTCs in a block without feedback, which served as the baseline. Subsequently, in a second block, we presented the same combinations of driving profiles and TTCs but informed the participants about their estimation accuracy after each trial. We withdrew the trial-by-trial feedback again in a third block. We expected that the estimated TTCs for accelerated approaches would follow a first-order approximation pattern in the first block, and would thus be similar to the estimated TTCs for constant-velocity approaches with the same speed and distance at the moment of estimation. The trial-by trial feedback in the second block was expected to help participants learn that they systematically overestimate the TTC in specific situations (i.e., for accelerated approaches), so that they would learn to distinguish between constant-velocity and accelerated vehicle approaches. We hypothesized for the second and third block, that the estimated TTCs would differ between the accelerated and constant-velocity approaches, because we expected the trial-by-trial feedback provided in the second block to help participants account for the acceleration of the vehicle in their TTC estimations.

## 2. Methods

### 2.1 TTC estimation task

**Fig 1:**
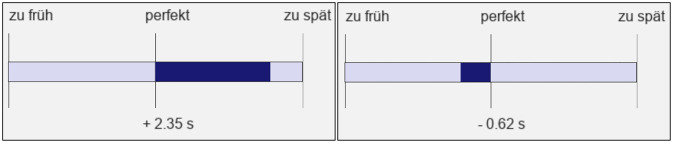
Example feedback panels about TTC estimation accuracy. The information was illustrated graphically (dark blue bar to the left (meaning you pressed the button “too early”; German: “zu früh”) or to the right (meaning you pressed the button “too late”; German: “zu spät”); the middle point shows reference to a perfect estimation (German: “perfekt”)) and also presented numerically (centrally below the bar). Left: TTC overestimation by 2.35 s. Right: TTC underestimation by 0.62 s. The bar was scaled proportionally only up to a deviation of 3.0 s between the estimated TTC and the veridical value.

To measure TTC estimation in a traffic scenario, we implemented a prediction-motion task [33] in a visual VR traffic simulation, as we did in a related article [8]. Per trial, a single vehicle approached the position of the participant in a virtual scene on the nearer lane of a two-lane road for 3.5 s. For the initial second, the vehicle always approached at a constant velocity. For the following 2.5 s, however, it either continued to travel with the same constant velocity or started to accelerate at a constant rate of 2.0 m/s². After 3.5 s, the vehicle disappeared from the display, as if passing behind an invisible occluder. Participants were tasked to estimate the moment at which they imagined that the vehicle would have arrived at their position if the vehicle had continued its motion. For their estimation, they were explicitly instructed to consider exactly the same velocity and acceleration of the vehicle as was presented before occlusion. The experimenter explained that some of the vehicles would travel at a constant velocity while others would accelerate. Participants indicated the estimated moment of arrival by pressing a button on a controller. The time interval between the occlusion and the button response served as estimated TTC in the respective trial. In the second of the three experimental blocks, participants received feedback about their estimation accuracy, which was presented for 2.5 s at the end of each trial. The feedback included both numerical information about the signed deviation of the estimated TTC from the presented TTC and a graphical illustration (Fig 1), and thus provided quantitative KR.

### 2.2 VR traffic simulation

**Fig 2:**
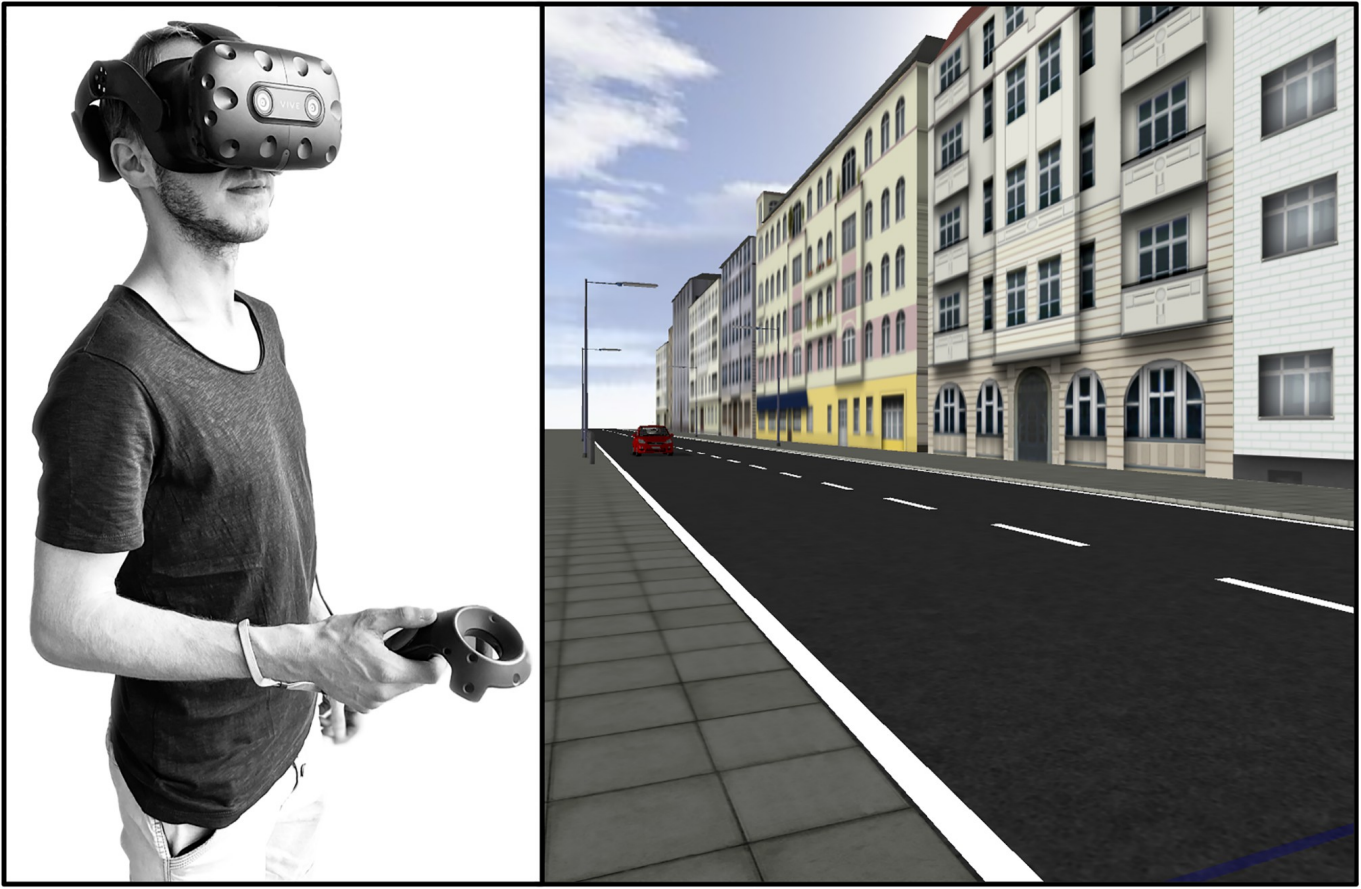
Participants wore an HMD (left) to observe the virtual traffic scene (right). They indicated their TTC estimates for the approaching car using the controller.

The visual simulation depicted an urban traffic scene, which was modeled after a two-lane road in Berlin (Eislebener Straße; 3D model retrieved from https://www.stadtentwicklung.berlin.de/planen/stadtmodelle/de/digitale_innenstadt/3d/index.shtml, provided by the Senate Department for Urban Development, Building and Housing of Berlin), with a red passenger car (modelled after a Mitsubishi Colt) approaching the participants from their left-hand side along the road. The participants observed the virtual scene from the sidewalk, 0.5 m away from the curb. 50 cm to the left of the observers’ virtual position, a blue line extended from the sidewalk across both lanes of the road, serving as an “arrival line” in the virtual environment. The participants wore a head-mounted display (HMD; HTC Vive Pro) that depicted the described scene (see Fig 2). The HMD head-tracking enabled the participants to explore the traffic scene and to track the vehicle with head movements. The interactive visual simulation was programmed using WorldViz’s VR software Vizard 7.

### 2.3 Experimental design

**Fig 3:**
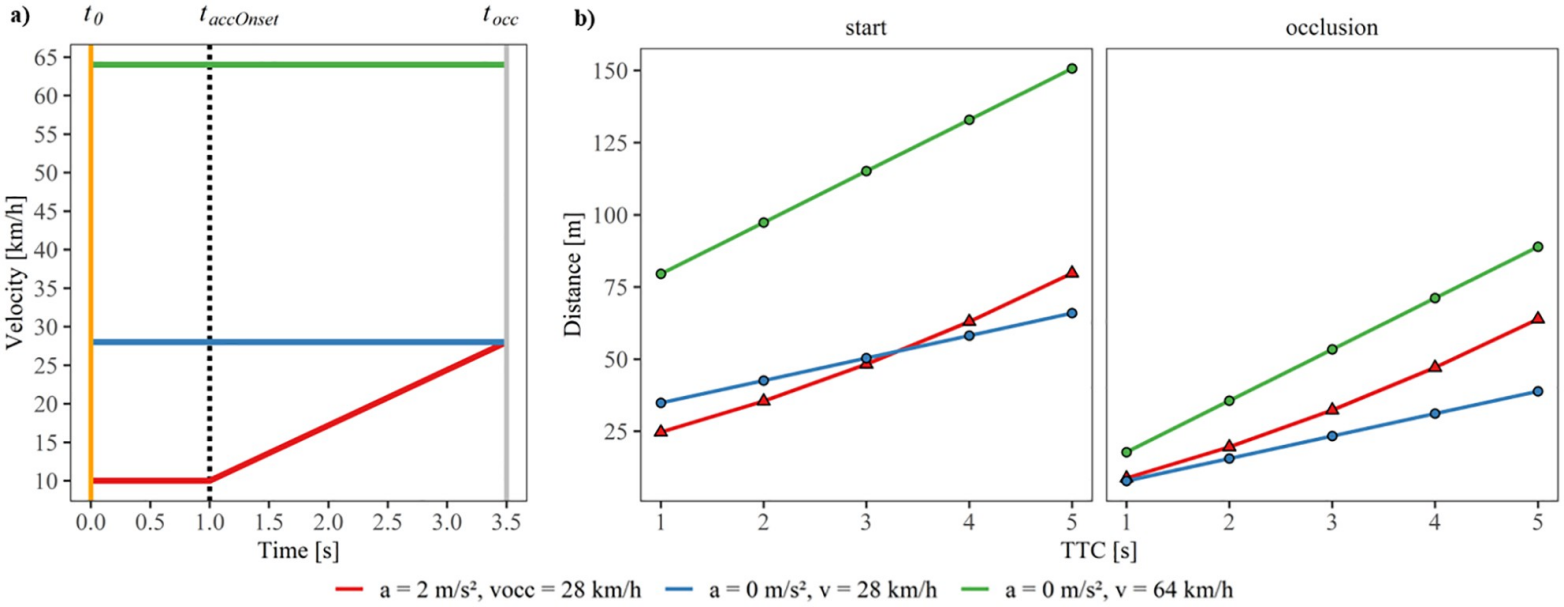
**a)** Velocity corresponding to the three different driving profiles across a trial. At the beginning of the trial (*t*_*0*_) for 1.0 s, the vehicle approached at a constant velocity and subsequently either started to accelerate at *t*_*accOnset*_ or continued to travel at the same constant velocity for 2.5 s. After the vehicle’s occlusion (*t*_*occ*,_), participants estimated the TTC for the vehicle. **b)** Vehicle distance as a function of actual TTC for the three different driving profiles at the start of the trial (left) and at occlusion (right). Red: accelerating vehicle (*a* = 2.0 m/s^2^) with a velocity at occlusion of 28 km/h. Blue: vehicle at a constant velocity of 28 km/h. Green: vehicle at a constant velocity of 64 km/h.

The experiment comprised vehicle approaches with three different *driving profiles*: two constant-velocity approaches of 28 km/h and 64 km/h, respectively, and an accelerated approach with an initial constant velocity of 10 km/h, an acceleration phase of 2.5 s during which the vehicle accelerated with 2.0 m/s² (similar to a previous study, [8]), and a velocity at occlusion (*v*_*occ*_) of 28 km/h. Thus, we presented both a constant-speed approach and an accelerated approach with a *v*_*occ*_ of 28 km/h, which made it possible to compare the TTC estimation for the accelerating vehicles with the TTC estimation for the constant-velocity vehicles (with exclusive first-order motion). Fig 3a shows the object velocity over the presentation time for the three driving profiles. Note that we chose the higher constant velocity mainly because it corresponded to farther vehicle distances at occlusion compared to the accelerated approach, so that a large distance at the start of the trial and at occlusion was not indicative of the vehicle acceleration (see Fig 3b). All driving profiles were presented with each of five different TTCs at occlusion (1.0, 2.0, 3.0, 4.0, and 5.0 s) in three experimental blocks, of which only the second block provided trial-by-trial feedback about the participants’ estimation accuracy (see Fig 1). The first (*pre feedback*) and third (*post feedback*) block each included 6 repetitions of each of the combinations of the 2 constant-velocity approaches and the 5 TTCs at occlusion, and 12 repetitions of the accelerated approach also at each of the 5 TTCs. Thus, they comprised the same number of constant-velocity and accelerated approaches and a total of 120 trials each. The second (*feedback*) block, in contrast, had 9 repetitions of the combinations including the constant-velocity approaches and 18 of the combinations including the accelerated ones (total of 180 trials). In total, all participants completed 420 experimental trials in a within-subjects design.

### 2.4 Procedure

Before the experiment, all participants had to pass two vision tests. We used the Titmus-test [34] to check for normal stereoscopic vision (criterion: at least 6 correct responses for the 9 presented binocular disparities of 800, 400, 200, 140, 100, 80, 60, 50 and 40 s of arc) and the Landolt ring optotype chart to screen for normal visual acuity (criterion: > 1.0). The distance between the displays of the HMD was adjusted according to the individual inter-pupillary distances. After participants were familiarized with the virtual traffic scene and the TTC estimation task in 16 training trials, they completed the experimental trials. After each block, the experimenter asked participants to take a break of ca. 10 min, but in addition, participants had the chance to take a break any time, since they initiated the start of each trial themselves. The experimenter controlled the participants’ well-being throughout the experiment using the Fast Motion Sickness Scale [35]. After experiment completion, the participants provided demographical information. The whole procedure with its pre-tests, TTC-estimation experiment, and final questionnaire lasted for about 3.5 hours.

### 2.5 Participants

20 participants (16 female, 4 male) with a mean age of 24.9 years (*SD* = 7.66 years) and (corrected-to) normal visual acuity and stereoscopic vision volunteered, completed the experiment, and received partial course credit. Before the testing, participants received information about the procedure and gave written informed consent regarding participation and data publication. The experimental procedure was in accordance with the principles outlined in the Declaration of Helsinki. The local ethics committee of the Johannes Gutenberg-University Mainz approved the study (approval number: 2019-JGU-psychEK-S011).

## 3. Results

### 3.1 Effects of block on TTC estimation patterns for accelerated and constant-velocity approaches at a velocity at occlusion of 28 km/h

**Fig 4:**
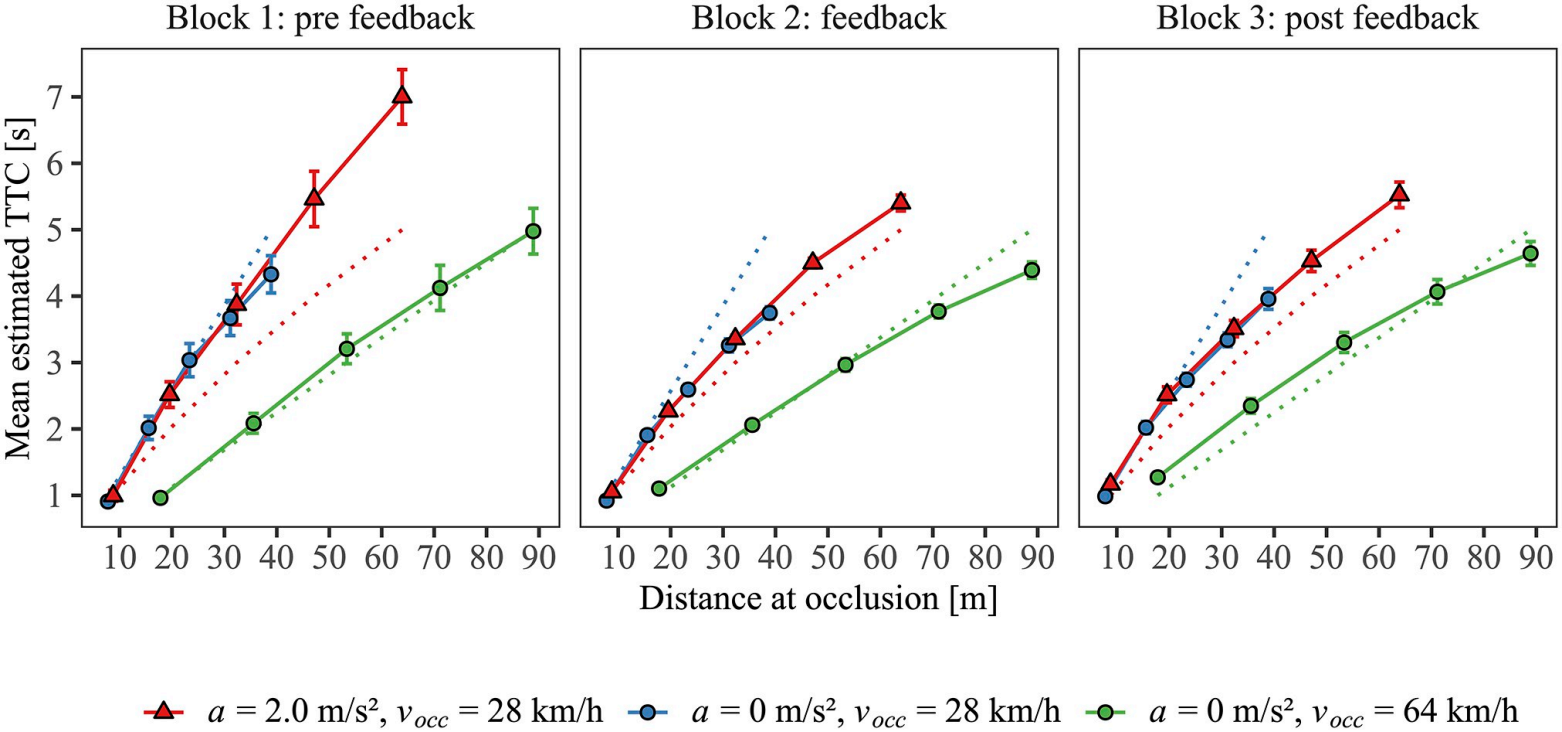
Mean estimated TTC as a function of vehicle distance at occlusion, driving profile and block (panels). Red triangles: accelerated approach with a velocity at occlusion of 28 km/h. Blue circles: constant-velocity approach at 28 km/h. Green circles: constant-velocity approach at 64 km/h. Dotted lines show the actual TTC for the respective driving profile. Error bars indicate ± 1 SE of the mean of the 20 individual values.

The main aim of the present study was to investigate if trial-by-trial feedback indicating the signed deviation between estimated and actual TTC at occlusion helps participants to account for acceleration in their estimations. Prior to the analyses, we applied a Tukey-criterion [36] to each combination of participant and experimental condition, so that extreme data points, which were either 3 interquartile ranges below the first or above the third quartile, were excluded (0.99% of a total of 8400 datapoints). For graphical illustrations, we aggregated the estimated TTCs per combination of participant and experimental condition. Fig 4 shows the mean estimated TTCs as a function of distance at occlusion (*D*_*occ*_) for a) the constant-velocity approach at 28 km/h (blue circles), b) the accelerated approach, for which the velocity at occlusion was also 28 km/h (red triangles), and c) the constant-velocity approach at 64 km/h (green circles). We first focus on the most interesting comparison between the constant-velocity and accelerated approach both at a velocity of 28 km/h (at occlusion) (comparison 1: a) vs. b)), and subsequently compare the two constant velocities (comparison 2: b) vs. c), see following section).

Regarding comparison 1, note that if participants made first-order estimations on the basis of the instantaneous velocity of the vehicle at occlusion, ignoring its acceleration, their estimated TTCs for the accelerated approach should be identical to the estimated TTCs for the constant-velocity approach with 28 km/h at the same distance at occlusion, because the first-order motion information (*D*_*occ*_*/v*_*occ*_) was identical for these two approaches. Put differently, the function relating *D*_*occ*_ and the mean estimated TTCs would be identical for the constant-speed and accelerating driving profiles with the same *v*_occ_, provided that participants performed a first-order estimation in both cases.

The left panel of Fig 4 shows that this was indeed the case in the first block (pre-feedback). The function relating *D*_*occ*_ to the mean estimated TTC for the accelerated approach (red line) was virtually identical to the function relating *D*_*occ*_ to the mean estimated TTC for the approach at a constant velocity of 28 km/h (blue line). The colored dotted lines in Fig 4 indicate the actual TTC for the respective driving profile at a specific distance. As expected, the mean estimated TTCs for the accelerated approach showed an overestimation of TTC that increased with the actual TTC (difference between solid and dotted lines). This is the typical first-order estimation pattern in visual TTC estimation for accelerating objects [37]. The mean estimated TTCs for the constant-velocity approach at 28 km/h were relatively close to the veridical values, showing only a small underestimation at the two longest TTCs.

In the second block, where trial-by-trial feedback was provided (middle panel of Fig 4), the mean estimated TTCs for both the accelerated approach and for the 28 km/h constant-velocity approach were shorter compared to the first block, particularly at the longer distances at occlusion. In other words, the function relating *D*_*occ*_ to the mean estimated TTC became more compressive in block 2 than in block 1. This distance-dependent shortening of the mean estimated TTCs in block 2 resulted in a reduced TTC overestimation for the accelerated approach, but caused a stronger underestimation of TTC for the constant-velocity approach, compared to block 1. Nonetheless, the function relating *D*_*occ*_ to the mean estimated TTC was still virtually identical for the accelerated approach and the constant-velocity approach at 28 km/h in block 2, as it was in block 1 (cf. red and blue solid lines in Fig 4). Thus, despite the trial-by-trial feedback provided in block 2, participants still made first-order TTC estimations. Descriptively, the same pattern as in block 2 was observed after withdrawal of the feedback in block 3 (post feedback; right panel of Fig 4).

Regardless of whether the TTC was overestimated or underestimated, the absolute TTC estimation error (defined as the absolute value of the difference between estimated and presented TTC on each trial) was–averaged across all driving profiles and final distances–descriptively smaller in block 2 (*M* = 0.57 s, *SD* = 0.10 s) and block 3 (*M* = 0.69 s, *SD* = 0.22 s) compared to the first block (*M* = 1.08 s, *SD* = 0.70 s). This reduction of the absolute TTC estimation error from block 1 to the following blocks was observed for each of the three driving profiles (see S1 Table). Thus, the shortening of the estimated TTCs adopted from block 2 on average caused a stronger underestimation at the longer TTCs in the 28 km/h-constant-speed condition (Fig 4) but did not result in impaired accuracy as measured by the absolute error.

**Fig 5:**
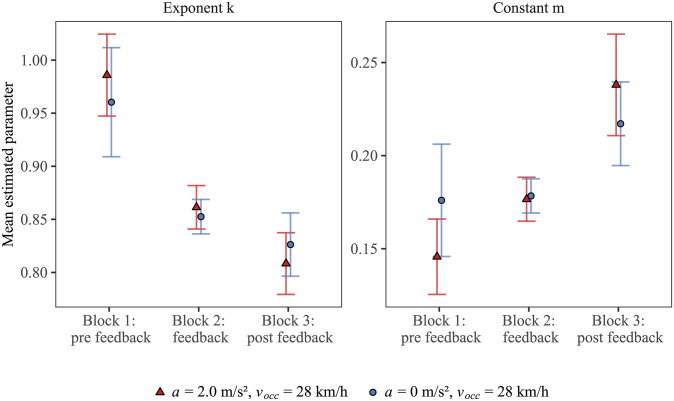
Mean estimated parameters *k* (left panel) and *m* (right panel) as a function of driving profile and block. Red triangles: accelerated approach with a velocity at occlusion of 28 km/h. Blue circles: constant-velocity approach at 28 km/h. Error bars indicate ± 1 SE of the mean of the 20 individual values.

To analyze the hypothesized effects of block on the mean estimated TTCs for the accelerated and constant-velocity approaches with *v*_*occ*_ = 28 km/h more formally, we fitted an effective observer model that is mainly based on vehicle distance at occlusion (see also [38]), and compared the estimated parameters between blocks. Fig 4 indicates that the mean estimated TTC was a compressive function of the distance at occlusion ($D_{occ}^{k}$, where *k* < 1 represents the compression) in all blocks and for all of the three driving profiles, which was also the case on the individual data level (see S1 Fig). For this reason, we fitted the function

$${TTC}_{est}={m∙D}_{occ}^{k},$$ (2)

separately to the estimated TTCs obtained in each combination of participant and block. The multiplicative constant *m* might be interpreted to represent the inverse of velocity in the equation. However, we do not intend to claim here that this effective model unequivocally represents the TTC estimation strategy participants actually used, because similar patterns of estimated TTCs would also result from, e.g., strategies based on different perceptual cues. Note that this statistical procedure was necessary to adequately compare the TTC estimations between the different driving profiles, because the factorial combinations of *v*_occ_, *a*, and TTC necessarily result in different vehicle distances at occlusion, and thus it was not possible to vary the occlusion distances independently of these other factors. Furthermore, the range of distances at occlusion for the accelerated and constant-velocity approaches were quite different. Thus, potential effects of driving profile on the fitted parameters could also be due to the systematic difference in distance ranges (see also S1 Appendix). Hence, we only included data points that corresponded to a distance of occlusion < 33 m (between 22 and 54 trials per combination), which ensured similar distance ranges for accelerated and constant-velocity-approaches in the fitting procedure. For the estimation of the two free parameters *k* and *m* in Eq 2, we used a non-linear least-squares approach (*R*-function *nls*()). Across participants, the goodness-of-fit of the individually fitted models was reasonably high (mean *R²* = 0.803, *SD* = 0.078). Fig 5 shows the mean estimated values for the parameters *m* and *k* for the constant-velocity and accelerated approach.

To test whether the function describing the individual TTC estimation patterns differed systematically between the accelerated and the constant-velocity approach at *v*_occ_ = 28 km/h and/or between the blocks, we conducted a multivariate analysis of variance (MANOVA) with the estimated values for the parameters *m* and *k* as the dependent variables, and *driving profile* (accelerated, constant-velocity approach with *v*_*occ*_ = 28 km/h) and *block* (block 1, block 2, block 3) as within-subjects factors. Because we had an equivalence hypothesis, *p*-values of < .20 were used as the cut-off for statistical significance. Pillai’s trace (*V*) was used as the multivariate test statistic. The fitted parameters for the accelerated and constant-speed approach at *v*_occ_ = 28 km/h did neither differ significantly across the three blocks, *V* = 0.01, *F*(2,18) = 0.06, *p* = .945, nor within the blocks as indicated by a non-significant interaction between *driving profile* and *block*, *V* = 0.14, *F*(4,76) = 1.46, *p* = .223. These results suggest that the patterns of estimated TTCs were on average rather similar between the accelerated and the constant-velocity approach at *v*_*occ*_ = 28 km/h. This is evident in the descriptive similarity of the functions relating *D*_*occ*_ and estimated TTC (Fig 4), consistently indicating a first-order TTC estimation strategy for accelerating vehicles in all three blocks. Finally, there was a significant effect of block on the mean estimated parameters, *V* = 0.60, *F*(4,76) = 8.13, *p* < .001. Fig 5 shows that both estimated parameters changed continuously over the course of the experiment, suggesting a successive modification of TTC estimation as a function of block. On average, the function relating *D*_*occ*_ and estimated TTCs depicted in Fig 4 was descriptively more compressive in the second and third block than in the first block, which suggests that participants shortened their estimated TTCs, in particular at large final vehicle distances, starting in the second (feedback) block.

### 3.2 TTC estimation patterns for the two constant-velocity approaches

The mean estimated TTCs for the constant-velocity approach at 64 km/h (green circles in Fig 4) were already relatively close to the presented TTCs in block 1. This pattern changed slightly in the two following blocks, showing shorter mean estimated TTCs at the largest *D*_occ_ in blocks 2 and 3, and additionally somewhat longer mean estimated TTCs at the smaller final distances in block 3. Note that the final vehicle distances from the observer at occlusion were much shorter for the approaches at the lower constant velocity than at the higher one. We refrained from fitting Eq 2 for the constant-velocity approaches because the large, systematic distance differences could potentially influence how the parameters *m* and *k* are fit. Nonetheless, the descriptive patterns (see Fig 4) suggest that compared to the first block, participants shortened their TTC estimations, on average, more strongly at larger compared to shorter final distances generally for all of the three driving profiles, so that the functions relating the final distance to estimated TTCs appeared to be more compressive in the subsequent two blocks (*feedback* and *post-feedback* block).

## 4. Discussion

In a simulated traffic scenario, we investigated TTC estimation for vehicles that either approached at a constant speed or accelerated positively, using a temporal prediction-motion task. As very consistently observed in the literature, untrained observers neglect to factor object acceleration into their estimated TTCs and often seem to use first-order estimation when only visual information is available [1–8]. In line with this, they predict the motion as if the accelerating approaching vehicle would continue to move at the instantaneous velocity it had at the moment of occlusion rather than continue its accelerated motion in a spatial prediction-motion task [37]. The main aim of our study was to investigate whether trial-by-trial feedback about the signed TTC estimation errors can reduce such systematic TTC estimation errors caused by first-order estimation and allow observers to use the correct second-order estimation strategy.

As expected, the estimated TTCs for accelerated approaches showed a clear first-order approximation pattern in the first block during which no feedback was provided. That is, accelerating vehicles were estimated to arrive at the collision point significantly later than they would have. In contrast, the TTCs of vehicles approaching at a constant velocity, be it at the slower (28 km/h) or at the faster (64 km/h) velocity, were judged fairly accurately. Most importantly, the function relating the mean estimated TTC and the distance between participant and vehicle at the moment of occlusion (*D*_*occ*_) was very similar for the accelerated approach and 28 km/h-constant-velocity approach, as if participants merely judged the TTC of the accelerating vehicle based on its final velocity (*v*_*occ*_ = 28 km/h) and distance (see Fig 4). Contrary to our hopes regarding the efficacy of training, this strong similarity between the pattern observed for the constant-speed and accelerated approach at the same velocity at occlusion was also evident in block 2 where trial-by-trial feedback was presented. In the final block without feedback (block 3), this pattern persisted. Hence, even during and after trial-by-trial feedback, observers were not able to properly consider second-order information; they failed to differentiate between constant-velocity and accelerated approaches. Nevertheless, the participants substantially changed their estimation strategy from block to block, but largely independent of second-order information. This is clear evidence that observers did process the feedback information.

### 4.1 Modification of the first-order TTC estimation strategy

Interestingly, the participants started to shorten their estimated TTCs particularly for larger distances of the vehicle at occlusion after block 1. This change in estimation strategy is characterized by the change in compression of the function relating *D*_*occ*_ to the mean estimated TTCs. As can be seen in Fig 4, the function became considerably more compressive in block 2 compared to block 1, and remained more compressive in block 3, underscoring that participants shortened their TTC estimations more strongly at larger compared to smaller final distances, starting in block 2. A potential explanation for this finding is that when the quantitative KR feedback was presented in block 2, particularly large estimation errors had become salient to the participants. Initially, these errors occurred particularly in the form of TTC overestimations for accelerating vehicles at large distances at the moment of occlusion (see Fig 4). The KR feedback in block 2 appears to have persistently weeded out particularly large overestimation errors by adjusting responses toward shorter estimated TTCs.

However, this change in TTC estimation observed between blocks was generally applied to both accelerating and constant-velocity vehicle approaches, although it would have been appropriate to distinguish by second-order information since the estimated TTCs for constant-velocity vehicles were already fairly accurate in block 1. The TTC estimation patterns remained similar between the accelerated approach and the constant-speed approach at 28 km/h, both on average (Fig 4) and at the individual level (see S1 Fig). This indicates that participants failed to account for acceleration during TTC estimation in blocks 2 and 3, but rather started to use an undifferentiated shortening of their TTC estimations whenever the vehicle was occluded at large distances. Put differently, it appears that the trial-by-trial feedback did not help participants to adopt a differential second-order estimation strategy, which would induce appropriate changes only in the estimations for accelerated approaches and prevent inappropriate changes in the estimations for constant-speed approaches. Instead, one could describe the data as showing that the feedback triggered a modified first-order strategy, which was applied in a similar manner to all types of driving profiles.

A more sophisticated second-order strategy for traffic scenarios has thus far only been found when the sound of an approaching vehicle (with internal combustion engine) was presented in addition to the visual object [7, 8]. The appropriate multisensory stimulus, including sound, helps to transform the first-order estimation strategy into a second-order strategy, virtually removing the TTC overestimation for accelerating vehicles, while leaving the TTC estimation for vehicles traveling at a constant velocity unchanged. One could interpret this as a trump of multisensory processing. However, participants could also have used a simple heuristic to shorten the TTC estimate in the presence of a vehicle sound signaling positive acceleration. Similarly, explicit visual acceleration signals could be an effective way to communicate positive acceleration to pedestrians. Implicit visual cues, in contrast, do not seem to enter the TTC estimation process, other than producing baseline shifts. Even visible cues to surface properties that should slow down objects moving on them, failed to produce differential effects [31]. Brenner et al. investigated whether the specific background surface, on which an object was horizontally moving, could help observers to anticipate the object’s deceleration and thereby help to intercept it with smaller errors. The data did not confirm this hypothesis. However, it remains unclear if the significance of the background surface texture had been explained to the participants. Hence, it seems that two conditions need to be fulfilled for observers to factor second-order information into their decisions: cues to acceleration have to be recognizable, and their meaning has to be explicit.

### 4.2 Why did feedback not help to consider acceleration in TTC estimation?

The inability of the trial-by-trial feedback to promote a second-order estimation strategy, may be due, at least in part, to the fact that observers are truly unable to visually detect acceleration [15–22]. If so, they would never visually differentiate between constant-speed and accelerated approaches. Then the change in TTC estimation strategy observed in block 2 (with trial-by-trial feedback) could be interpreted as a rational reaction in the face of an inability to properly differentiate among second-order and first-order (i.e., accelerated and constant-velocity approaches) approaches. If the observer is repeatedly told that she or he hit the response button too late, particularly when the vehicle was relatively far away at occlusion, adjustments toward shorter estimated TTCs are a reasonable strategy. Apparently, this was applied to all approaches, not just to the accelerated approaches where it would have only been appropriate. This unspecific strategy was applied such that it made the estimated TTCs of accelerating vehicles more accurate but at the same time introduced a stronger underestimation of TTC for the 28-km/h constant-velocity trials.

In a recent study [39], with a similar setup and similar accelerated trajectories, participants detected the acceleration of an object, or at least recognized a change in velocity during presentation, with an accuracy of approximately 75% in a condition providing visual-only presentations of the approaching vehicles. This was well above the guessing rate of 0.5 but far from perfect acceleration detection. Note that the presentation duration after acceleration onset was 500 ms longer than in the present study, so that the shorter presentation duration might have further reduced the acceleration detection accuracy in the present experiment. This appears particularly plausible if observers perceive acceleration only indirectly by relative change between initial and final velocity, as this comparison is systematically influenced by the presentation duration (e.g., [22, 40]). In light of the results obtained by Oberfeld et. al. [40, Experiment 3] it is unlikely that participants were completely unable to detect when a vehicle was accelerating. In addition, Pekkanen et al. [41] modeled road-crossing decisions and reported that the pedestrians did use deceleration cues in their decisions, that is, they were more likely to cross the road when vehicles yielded. This, again, would require participants to have detected the (negative) acceleration, or at least correlated cues, such as a threshold velocity value as a trigger for crossing behavior. Since the study used only negative accelerations, it is unclear how the results translate to scenarios with positive acceleration. Thus, in future experiments, it would be interesting to study the relation between acceleration detection accuracy and TTC estimation patterns for accelerating vehicles on the individual level, to explore to what extent the first-order estimation for accelerating vehicles could be due to poor acceleration detection sensitivity.

### 4.3 Practical implications

Although our results indicate that trial-by-trial KR feedback did not enable participants to use second-order motion information, from a traffic-safety perspective, the training had a beneficial effect–at least regarding the TTC estimation for the presented driving profiles. During the course of the experiment, the TTC overestimations for the accelerating vehicles observed in block 1 were strongly reduced, and for the constant speed conditions, a tendency towards an underestimation of TTC was observed. Although underestimations constitute errors in TTC estimation, they are not associated with risky street-crossing decisions, because they would effectively result in more cautions crossing decisions. The accuracy in terms of the absolute error also improved during the course of the experiment. Nonetheless, participants only modified their first-order estimation strategy for the accelerating vehicles, starting in block 2 where KR feedback was provided; they did not adopt a second-order estimation strategy. At a higher acceleration rate, the specific and distance-dependent shortening of the first-order estimations adopted in block 2 for an acceleration rate of with 2.0 m/s² might therefore not be sufficient so that (risky) TTC overestimation might continue to occur. Accordingly, training with KR feedback would likely need to include sufficiently variable acceleration levels to be useful for real-world road use. In contrast, recent research showed that explicitly communicating the presence/absence or even the strength of the acceleration, e.g., by presenting the vehicle sound, allows for second-order estimation [7, 8] and could thus be more efficient to counteract pedestrians’ erroneous TTC estimation for accelerating vehicles than training with KR feedback.

### 4.4 Limitations

As plausible as the blanket processing of the feedback (block 2) may be, we cannot causally attribute the changes in the TTC estimations observed between block 1 and blocks 2 and 3 to the trial-by-trial feedback, because our study did not include a control group that received no feedback in block 2. Experiments on perceptual learning have demonstrated that changes in performance also happen in the absence of feedback (“training without trainer”) [28]. Also, block feedback can be as effective as trial-by-trial feedback [28]. Thus, additional experiments would be necessary to confirm causal evidence for an effect of the feedback.

A second limitation of our experimental design was that the distances at occlusion were not matched between the different driving profiles. In TTC experiments, we cannot control all parameters at the same time. We had chosen to match the TTCs across driving profiles rather than match final distance at occlusion, in order to present TTCs relevant for street-crossing decisions in all conditions. As a consequence, the effect of block on the estimated TTCs of the 28-km/h constant-velocity approaches at larger final distances had to be inferred from the fitted functions, because for this driving profile the maximal distance at occlusion was considerably shorter than for the two other conditions. This might partly explain the differences in the estimated parameters of Eq 2 between the constant-velocity approach at 28 km/h and the accelerated approach. Additional data in a design that matches distance at occlusion–at the expense of matched TTCs—would be desirable to confirm the TTC estimation patterns.

Another limitation of our experiment is that block 2, in which the trial-by-trial KR feedback was presented, was relatively short, presenting 90 trials for the accelerating vehicle and 45 trials each for the constant-speed approaches. It thus remains to be shown if quantitative KR feedback received during a considerably longer training phase of several hours or even weeks would have the desired effect of enabling participants to spontaneously use second-order motion information in their TTC estimations.

Finally, we only presented an acceleration rate of 2.0 m/s² in the present experiment. As described above, the feedback might have motivated participants to shorten their estimations to an extent that compensates TTC overestimations for the accelerated approach at this specific acceleration rate. However, Eq 1 shows that TTC overestimation increases with increasing acceleration rates. It could thus be that the modified estimation strategy observed for the accelerated approaches in this study is limited to the presented acceleration rate, so that observers may still substantially overestimate TTCs of vehicles at higher acceleration rates. Against this background, the reduction of TTC overestimation observed in this study should be treated with caution when inferring benefits of feedback training for traffic safety.

## 5. Conclusion

Observers perform notoriously poorly when it comes to the anticipation of the collision time (TTC) of an object that approaches in a positively accelerated fashion. That is, they neglect the acceleration (second-order) information and judge the TTC for an accelerating object as if it were traveling at a constant velocity, which results in TTC overestimation. We provided ample decision time (2500 ms) in combination with trial-by-trial knowledge of results (KR) feedback, to maximize the chance that observers would learn to properly differentiate between constant-velocity and accelerated approaches. However, this was not the case. Observers were unable to exploit the feedback to modulate their estimates on the basis of second-order information. Instead, they seemed to uniformly apply a downwards correction to their estimated TTCs for both constant-velocity and accelerating vehicles. The results of the present study reveal the severity and robustness of the estimation errors committed when judging the TTC for visually presented accelerating vehicles. Trial-by-trial KR feedback about the signed deviation of the estimated from the actual TTC failed in making observers account for second-order information for accelerated approaches. From a practical point of view, a safety training program including feedback about TTC estimation accuracy does not promise to be an effective tool to help pedestrians consider a vehicle’s acceleration adequately, when deciding whether or not to cross the road in front of the vehicle.

## Supporting information

S1 Appendix Results of fitting Eq 2 without restricting the distance ranges of the accelerated and constant-velocity approaches with *v*_*occ*_ = 28 km/h. (DOCX)

S1 Table Descriptive statistics of the absolute TTC estimation error. (DOCX)

S1 Fig Mean estimated TTCs of each participant. (DOCX)

## References

[1] Benguigui N, Ripoll H, Broderick MP. Time-to-Contact Estimation of Accelerated Stimuli Is Based on First-Order Information. Journal of Experimental Psychology: Human Perception and Performance. 2003;29(6):1083–101. doi: 10.1037/0096-1523.29.6.1083 14640832

[2] Benguigui N, Bennett SJ. Ocular pursuit and the estimation of time-to-contact with accelerating objects in prediction motion are controlled independently based on first-order estimates. Exp Brain Res. 2010 Apr;202(2):327–39. doi: 10.1007/s00221-009-2139-0 20039024

[3] Kaiser MK, Hecht H. Time-to-passage judgments in nonconstant optical flow fields. Perception & Psychophysics. 1995 Jan;57(6):817–25. doi: 10.3758/bf03206797 7651806

[4] Kreyenmeier P, Kämmer L, Fooken J, Spering M. Humans Can Track But Fail to Predict Accelerating Objects. eNeuro. 2022 Sep;9(5):ENEURO.0185-22.2022. doi: 10.1523/ENEURO.0185-22.2022 36635938 PMC9469915

[5] Lee DN, Young DS, Reddish PE, Lough S, Clayton TMH. Visual Timing in Hitting An Accelerating Ball. The Quarterly Journal of Experimental Psychology Section A. 1983 May;35(2):333–46. doi: 10.1080/14640748308402138 6571315

[6] López-Moliner J, Maiche A, Estaún S. Perception of acceleration in motion-in-depth with only monocular and both monocular and binocular information. Psicológica. 2003;24:93–108.

[7] Wessels M, Kröling S, Oberfeld D. Audiovisual time-to-collision estimation for accelerating vehicles: The acoustic signature of electric vehicles impairs pedestrians’ judgments. Transportation Research Part F: Traffic Psychology and Behaviour. 2022 Nov;91:191–212.

[8] Wessels M, Zähme C, Oberfeld D. Auditory Information Improves Time-to-collision Estimation for Accelerating Vehicles. Curr Psychol [Internet]. 2022 Jul 15 [cited 2022 Oct 6]; https://link.springer.com/10.1007/s12144-022-03375-6

[9] Petzoldt T. On the relationship between pedestrian gap acceptance and time to arrival estimates. Accident Analysis & Prevention. 2014 Nov;72:127–33. doi: 10.1016/j.aap.2014.06.019 25035969

[10] Dommes A, Cavallo V, Oxley J. Functional declines as predictors of risky street-crossing decisions in older pedestrians. Accident Analysis & Prevention. 2013 Oct;59:135–43. doi: 10.1016/j.aap.2013.05.017 23792612

[11] Dommes A, Cavallo V. The role of perceptual, cognitive, and motor abilities in street-crossing decisions of young and older pedestrians. Ophthalmic and Physiological Optics. 2011;31(3):292–301. doi: 10.1111/j.1475-1313.2011.00835.x 21470273

[12] Feldstein IT, Dyszak GN. Road crossing decisions in real and virtual environments: A comparative study on simulator validity. Accident Analysis & Prevention. 2020 Mar;137:105356. doi: 10.1016/j.aap.2019.105356 32059135

[13] Summala H. Towards Understanding Motivational and Emotional Factors in Driver Behaviour: Comfort Through Satisficing. In: Cacciabue PC, editor. Modelling Driver Behaviour in Automotive Environments [Internet]. London: Springer London; 2007 [cited 2023 May 9]. p. 189–207. http://link.springer.com/10.1007/978-1-84628-618-6_11

[14] Tresilian JR. Perceptual and cognitive processes in time-to-contact estimation: Analysis of prediction-motion and relative judgment tasks. Perception & Psychophysics. 1995 Jan;57(2):231–45. doi: 10.3758/bf03206510 7885822

[15] Calderone JB, Kaiser MK. Visual acceleration detection: Effect of sign and motion orientation. Perception & Psychophysics. 1989 Sep;45(5):391–4. doi: 10.3758/bf03210711 2726400

[16] Gottsdanker R, Frick JW, Lockard R. Identifying the acceleration of visual targets. British Journal of Psychology. 1961;52(1):31–42. doi: 10.1111/j.2044-8295.1961.tb00765.x 13707460

[17] Mueller AS, González EG, McNorgan C, Steinbach MJ, Timney B. Aperture extent and stimulus speed affect the perception of visual acceleration. Exp Brain Res. 2017 Mar;235(3):743–52. doi: 10.1007/s00221-016-4824-0 27866263

[18] Runeson S. Constant velocity? Not perceived as such. Psychol Res. 1974;37(1):3–23. doi: 10.1007/BF00309076 4449922

[19] Snowden RJ, Braddick OJ. The temporal integration and resolution of velocity signals. Vision Research. 1991 Jan;31(5):907–14. doi: 10.1016/0042-6989(91)90156-y 2035273

[20] Trewhella J, Edwards M, Ibbotson MR. Sensitivity to the acceleration of looming stimuli. Clin Exp Ophthalmol. 2003 Jun;31(3):258–61. doi: 10.1046/j.1442-9071.2003.00641.x 12786780

[21] Watamaniuk SNJ, Heinen SJ. Perceptual and oculomotor evidence of limitations on processing accelerating motion. Journal of Vision. 2003 Nov 21;3(11):5. doi: 10.1167/3.11.5 14765954

[22] Werkhoven P, Snippe HP, Alexander T. Visual processing of optic acceleration. Vision Research. 1992 Dec;32(12):2313–29. doi: 10.1016/0042-6989(92)90095-z 1288008

[23] Jörges B, López-Moliner J. Gravity as a Strong Prior: Implications for Perception and Action. Front Hum Neurosci. 2017 Apr 28;11:203. doi: 10.3389/fnhum.2017.00203 28503140 PMC5408029

[24] Ceccarelli F, La Scaleia B, Russo M, Cesqui B, Gravano S, Mezzetti M, et al. Rolling Motion Along an Incline: Visual Sensitivity to the Relation Between Acceleration and Slope. Front Neurosci. 2018 Jun 22;12:406. doi: 10.3389/fnins.2018.00406 29988401 PMC6023988

[25] Senot P, Zago M, Lacquaniti F, McIntyre J. Anticipating the Effects of Gravity When Intercepting Moving Objects: Differentiating Up and Down Based on Nonvisual Cues. Journal of Neurophysiology. 2005 Dec;94(6):4471–80. doi: 10.1152/jn.00527.2005 16120661

[26] Hecht H, Kaiser MK, Banks MS. Gravitational acceleration as a cue for absolute size and distance? Perception & Psychophysics. 1996 Jan;58(7):1066–75. doi: 10.3758/bf03206833 8920842

[27] Braly AM. Direct Learning for Time-to-Collision Judgments of Approaching Objects: The Role of Fractal 1/f Noise in Exploration. [Houston, Texas]: Rice University; 2020.

[28] Herzog MH, Fahle M. The role of feedback in learning a vernier discrimination task. Vision Research. 1997 Aug;37(15):2133–41. doi: 10.1016/s0042-6989(97)00043-6 9327060

[29] Jacobs DM, Michaels CF, Runeson S. Learning to perceive the relative mass of colliding balls: The effects of ratio scaling and feedback. Perception & Psychophysics. 2000 Oct;62(7):1332–40. doi: 10.3758/bf03212135 11143445

[30] Michaels CF, de Vries MM. Higher order and lower order variables in the visual perception of relative pulling force. Journal of Experimental Psychology: Human Perception and Performance. 1998;24:526–46. doi: 10.1037//0096-1523.24.2.526 9554096

[31] Brenner E, Rodriguez IA, Muñoz VE, Schootemeijer S, Mahieu Y, Veerkamp K, et al. How Can People Be so Good at Intercepting Accelerating Objects if They Are so Poor at Visually Judging Acceleration? i-Perception. 2016 Feb 1;7(1):204166951562431. doi: 10.1177/2041669515624317 27482367 PMC4954742

[32] Brenner E, de la Malla C, Smeets JBJ. Tapping on a target: dealing with uncertainty about its position and motion. Exp Brain Res. 2023 Jan;241(1):81–104. doi: 10.1007/s00221-022-06503-7 36371477 PMC9870842

[33] Schiff W, Detwiler ML. Information used in judging impending collision. Perception. 1979;8(6):647–58. doi: 10.1068/p080647 530806

[34] Bennett AG, Rabbetts RB. Clinical visual optics. 3rd ed. Oxford: Butterworth-Heinemann; 1998.

[35] Keshavarz B, Hecht H. Validating an Efficient Method to Quantify Motion Sickness. Hum Factors. 2011 Aug;53(4):415–26. doi: 10.1177/0018720811403736 21901938

[36] Tukey JW. Exploratory Data Analysis. Reading, Mass: Addison-Wesley Pub Co. 1977;

[37] Bennett SJ, Benguigui N. Spatial Estimation of Accelerated Stimuli Is Based on a Linear Extrapolation of First-Order Information. Experimental Psychology. 2016 Mar;63(2):98–106. doi: 10.1027/1618-3169/a000318 27221600

[38] Bernhard C, Oberfeld D, Hecht H. Rear-view perception in driving: Distance information is privileged in the selection of safe gaps. Transportation Research Part F: Traffic Psychology and Behaviour. 2022 Apr;86:263–80.

[39] Oberfeld D, Wessels M, Kröling S. Risiko hohe Beschleunigung? Straßenquerungsverhalten von Fußgänger:innen in Interaktion mit E-Fahrzeugen (mit und ohne AVAS) im Vergleich zu Verbrennern [Internet]. Berlin: Gesamtverband der Deutschen Versicherungswirtschaft e.V.; 2021. https://www.udv.de/resource/blob/84078/22741be085f0aa88579fe0c7362867c6/76-risiko-hohe-beschleunigung-d-data.pdf

[40] Brouwer AM, Brenner E, Smeets JBJ. Perception of acceleration with short presentation times: Can acceleration be used in interception? Perception & Psychophysics. 2002 Oct;64(7):1160–8. doi: 10.3758/bf03194764 12489669

[41] Pekkanen J, Giles OT, Lee YM, Madigan R, Daimon T, Merat N, et al. Variable-Drift Diffusion Models of Pedestrian Road-Crossing Decisions. Comput Brain Behav. 2022 Mar;5(1):60–80.

