# Correction: Trial-by-trial feedback fails to improve the consideration of acceleration in visual time-to-collision estimation

**DOI:** 10.1371/journal.pone.0315441

**Published:** 2024-12-05

**Authors:** Marlene Wessels, Heiko Hecht, Thirsa Huisman, Daniel Oberfeld

There are errors in the Author Contributions. The correct contributions are:

**Conceptualization:** Marlene Wessels, Heiko Hecht, Thirsa Huisman, Daniel Oberfeld

**Formal analysis:** Marlene Wessels, Daniel Oberfeld

**Funding acquisition:** Daniel Oberfeld

**Investigation:** Marlene Wessels

**Visualization:** Marlene Wessels,

**Writing–original draft:** Marlene Wessels, Daniel Oberfeld

**Writing–review & editing:** Marlene Wessels, Heiko Hecht, Thirsa Huisman, Daniel Oberfeld
