# Supplementary material for: Trial-by-trial feedback fails to improve the consideration of acceleration in visual time-to-collision estimation
Source: PLoS One. 2023 Aug 2;18(8):e0288206. doi: 10.1371/journal.pone.0288206 (PMC10395816; doi:10.1371/journal.pone.0288206)
Supplement: S1 Appendix — (DOCX) [file pone.0288206.s001.docx]

## S1 Appendix. Results of fitting Eq. 2 without restricting the distance ranges of the accelerated and constant-velocity approaches with *v_occ_* = 28 km/h.

An anonymous reviewer pointed out that the different distance ranges of the accelerated and constant-velocity approaches (both with *v_occ_* = 28 km/h) could substantially influence the fitting procedure described in the Results section. We therefore only included data points that corresponded to a distance of occlusion < 33 m in the main analysis, ensuring similar distance ranges for both driving profiles. Nonetheless, we report here the results of the fitting procedure without restricting the distance ranges of the accelerated and constant-velocity approaches with *v_occ_* = 28 km/h, which should be treated with caution because of the aforementioned reason.

We conducted two repeated-measures analyses of variance (ANOVAs) with a univariate approach and Huynh-Feldt correction for the degrees of freedom, separately for each of the two estimated parameters. Standard *p*-values of < .050 were used as cut-off for statistical significance. Across the three blocks, the estimated exponent *k* did not differ significantly between the accelerated and constant-speed approach at *v*_occ_ = 28 km/h, *F*(1,19) = 1.71, *p* = .206, *η^2^_p_* = .08. There was also no significant effect of driving profile on the estimated parameter *m, F*(1,19) = 3.45, *p* = .079, *η^2^_p_* = .15. The effect of block on the mean estimated values for the exponent *k* was significant, *F*(2,38) = 19.05, *p* < .001, *η^2^_p_* = .50. There was also a significant effect of block on the estimated values for the constant *m, F*(2,38) = 4.27, *p* = .025, *η^2^_p_* = .18. Finally, the block × driving profile interaction was significant for both of the estimated parameters (parameter *k*: *F*(2,38) = 5.60, *p* = .011, *η^2^_p_* = .23; parameter *m*: *F*(2,38) = 6.24, *p* = .005, *η^2^_p_* = .25). Please note that 1) effects involving the driving profile could be due to the confound between driving profile and distance range, and 2) no significant main effect of driving profile/no significant block × driving profile interaction effect was found in the analysis based on restricted distance ranges (< 33m) described in the Results section.
