# Supplementary material for: Trial-by-trial feedback fails to improve the consideration of acceleration in visual time-to-collision estimation
Source: PLoS One. 2023 Aug 2;18(8):e0288206. doi: 10.1371/journal.pone.0288206 (PMC10395816; doi:10.1371/journal.pone.0288206)
Supplement: S1 Table — (DOCX) [file pone.0288206.s002.docx]

## S1 Table. Descriptive statistics of the absolute TTC estimation error.

S1 Table: Descriptive statistics of the absolute TTC estimation error (defined as the absolute value of the difference between estimated and presented TTC on each trial) for each of the driving profiles per block. Displayed are the mean and standard deviation (*SD*) in s.

|  |  | *Mean (s)* | *SD (s)* | |
| --- | --- | --- | --- | --- |
| 28 km/h constant-velocity approach | Block 1 (pre feedback) | 0.82 | | 0.56 |
|  | Block 2 (feedback) | 0.65 | | 0.14 |
|  | Block 3 (post feedback) | 0.63 | | 0.19 |
| 64 km/h constant-velocity approach | Block 1 (pre feedback) | 0.89 | | 0.56 |
|  | Block 2 (feedback) | 0.55 | | 0.12 |
|  | Block 3 (post feedback) | 0.66 | | 0.28 |
| Accelerated approach | Block 1 (pre feedback) | 1.30 | | 0.93 |
|  | Block 2 (feedback) | 0.55 | | 0.15 |
|  | Block 3 (post feedback) | 0.74 | | 0.31 |
