# Supplementary material for: Trial-by-trial feedback fails to improve the consideration of acceleration in visual time-to-collision estimation
Source: PLoS One. 2023 Aug 2;18(8):e0288206. doi: 10.1371/journal.pone.0288206 (PMC10395816; doi:10.1371/journal.pone.0288206)
Supplement: S1 Fig — (DOCX) [file pone.0288206.s003.docx]

## S1 Fig. Mean estimated TTCs of each participant.

| 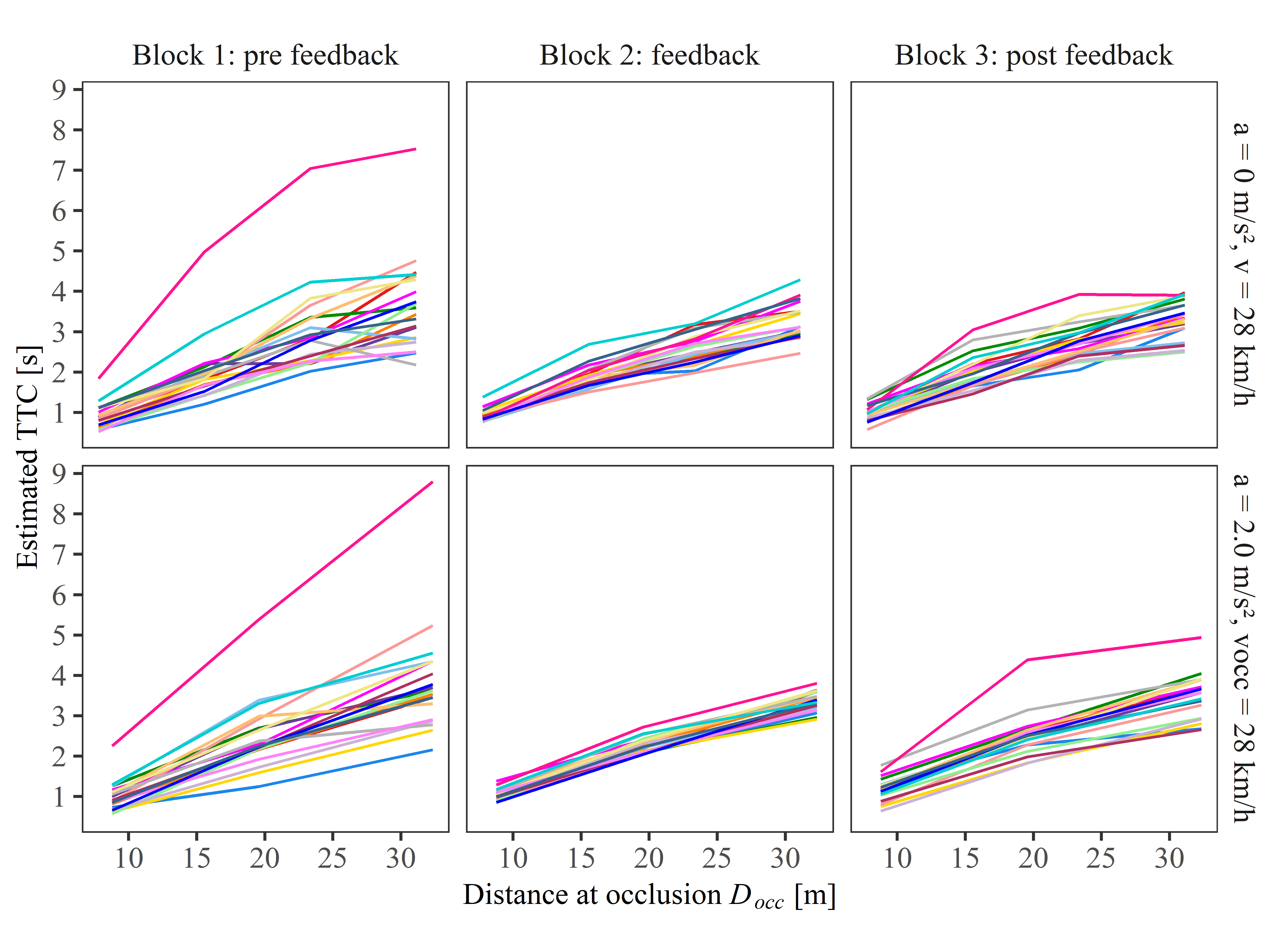 |
| --- |
| S1 Fig: Mean estimated TTCs of each participant (color-coded) as a function of the vehicle distances at occlusion *D_occ_* (x-axis), for the two driving profiles (upper row: constant velocity; lower row: accelerated approach) and the three experimental blocks (columns). Note that this figure is based on the data subset used for the fitting procedure described in the Results section (*D_occ_* < 33 m). |
